# Impaired trunk control and its relationship with balance, functional mobility, and disease severity in patients with cervical dystonia

**DOI:** 10.55730/1300-0144.5597

**Published:** 2022-11-30

**Authors:** Fatih SÖKE, Nigar Esra ERKOÇ ATAOĞLU, Mehmet Fevzi ÖZTEKİN, Bilge KOÇER, Selda KARAKOÇ, Çağrı GÜLŞEN, Selim Selçuk ÇOMOĞLU, Ayşe BORA TOKÇAER

**Affiliations:** 1 Department of Physiotherapy and Rehabilitation, Gülhane Faculty of Physiotherapy and Rehabilitation, University of Health Sciences, Ankara, Turkey; 2 Department of Neurology, Faculty of Medicine, Gazi University, Ankara, Turkey; 3 Department of Neurology, Dışkapı Yıldırım Beyazıt Teaching and Research Hospital, University of Health Sciences, Ankara, Turkey; 4 Department of Physiotherapy and Rehabilitation, Gülhane Institute of Health Science, University of Health Sciences, Ankara, Turkey; 5 Department of Physiotherapy and Rehabilitation, Faculty of Health Sciences, Gazi University, Ankara, Turkey

**Keywords:** Cervical dystonia, trunk control, balance, functional mobility, disease severity

## Abstract

**Background/aim:**

Impaired trunk control is common in neurological disorders; however, trunk control has not been examined in patients with cervical dystonia (CD). Therefore, the primary aim was to compare trunk control between patients with CD and healthy people. The secondary aim was to investigate the relationship between trunk control and balance, functional mobility, and disease severity in patients with CD.

**Materials and methods:**

This cross-sectional study included 32 patients with CD and 32 healthy people. Trunk control was compared using the trunk impairment scale (TIS) that consists of three subscales: static sitting balance, dynamic sitting balance, and trunk coordination between two groups. Balance was assessed using Berg Balance Scale, four square step test, and one-leg stance test. The Timed Up and Go Test was measured to determine functional mobility. Toronto Western Spasmodic Torticollis Rating Scale was used to evaluate disease severity.

**Results:**

Patients with CD demonstrated worse performance on the TIS-total with TIS-dynamic sitting subscale and TIS-trunk coordination subscale (p < 0.001, p < 0.001, and p < 0.001), except for TIS-static sitting subscale (p = 0.078) compared to healthy people. TIS-total scores had moderate to strong correlations with balance, functional mobility, and disease severity (range r between 0.786 and 0.536, p < 0.05 for all). There was no correlation between TIS-total scores and disease severity (p = 0.102).

**Conclusion:**

Patients with CD had impaired trunk control, especially in dynamic sitting balance and trunk coordination. Impaired trunk control was also associated with balance and functional mobility but not disease severity. These findings suggest that trunk control deficits should receive attention in the assessment and treatment of patients with CD.

## 1. Introduction

Cervical dystonia (CD) is a chronic neurologic disease characterized by sustained and involuntary contractions of the neck muscles that cause nonfunctional neck and head postures [1,2]. The phenomenology of CD is more complex, consisting of both the impairment of voluntary movements and the presence of involuntary motions, such as tremor, jerky, and spasm [3]. CD leads to abnormalities in many brain areas, including basal ganglia, cerebral cortex, cerebellum, and vestibular pathways [4–6].

Dystonia can adversely affect movement control and balance even when it does not have a direct impact on the limbs or trunk such as CD [7]. Compared to healthy people, patients with CD have decreased balance and functional mobility performance [8]. Control of trunk movements is required to maintain body equilibrium and achieve smooth locomotion [9–11]. Trunk control is considered as the main contributor to balance and functional mobility [9, 11–13]. Impaired trunk control has been reported in multiple sclerosis [14], stroke [15], and Parkinson’s disease (PD) [16]. The assessment of trunk control; thus, is of great importance in neurologic populations. However, to our knowledge, no study has investigated trunk control and its associations with balance, functional mobility, and disease severity in patients with CD. Therefore, the primary aim of this study was to compare trunk control between patients with CD and healthy people. The secondary aim was to explore the relationship between trunk control and balance, functional mobility, and disease severity in patients with CD.

## 2. Materials and methods

### 2.1. Study design

This cross-sectional study was performed at the Department of Neurology of Gazi University, between May and July 2022. This study was confirmed by Gazi University Clinical Research Ethics Committee (approval number: 351), and was in accordance with the Declaration of Helsinki. All participants signed informed consent prior to participation in the study.

### 2.2. Participants

No study has examined the trunk impairment scale in patients with CD up to now; therefore, the sample size was provided with a power calculation based on the differences in trunk impairment scale (TIS) score between multiple sclerosis as a neurological disease and healthy people. A previous study reported that patients with multiple sclerosis had 20.29 ± 4.88 points while healthy people had 23.00 ± 0.0 points on the TIS [17]. To detect a difference of 2.71 points in TIS scores between groups with an effect size of 0.78 with %80 power at a 5% significance level, at least 27 participants were required per group.

Patients, who were diagnosed with CD, were invited by a neurologist in the present study. Inclusion criteria were at least 18 years of age, able to walk independently with or without a walking aid, and at least 3 months since the last injection or immediately prior to a new injection of botulinum toxin to minimize the clinical effect of the injection. Exclusion criteria were other neurologic disorders, antalgic/pathologic gait pattern, secondary or hereditary forms of dystonia, dystonia in other body parts than the neck, and any other conditions that affect trunk, balance, and gait. Age- and sex-matched healthy participants with the same exclusion criteria were included as the control group in the study.

### 2.3. Procedures

Demographic and clinical characteristics of all participants were recorded. Patients with CD were assessed with the TIS, Berg Balance Scale (BBS), four square step test (FSST), one-leg stance test (OLST), timed up and go test (TUG), and Toronto Western Spasmodic Torticollis Rating Scale (TWSTRS), respectively. Three different assessments, which were the BBS, FSST, and OLST, were used to measure the functional, dynamic and static components of balance, respectively. To compare patients with CD, healthy people only performed the TIS.

The TIS is used to assess trunk control, and has three subscales including static sitting balance, dynamic sitting balance, and trunk coordination. The maximal score of the static and dynamic sitting balance and trunk coordination is 7, 10, and 6, respectively. The total score of TIS ranges between 0 and 23. Higher score indicates better trunk control [18]. The test-retest and interrater reliability are high for the TIS in stroke [intraclass correlation coefficient (ICC) = 0.96 and ICC = 0.99, respectively] [19].

The BBS evaluates functional balance, and has 14 items related to daily living activities. Each item is scored from 0 to 4. The total score is between 0 and 56, with higher scores representing better functional balance [20]. The BBS has high test-retest reliability for patients with PD (ICC) = 0.94 [21].

The FSST is a clinical test assessing dynamic balance performance. Participants are asked to step forward, backward, and sideways over canes on the floor as rapidly as possible. The completion time is recorded, with lower time demonstrating better dynamic balance [22]. For patients with PD, the FSST demonstrates high interrater and good test-retest reliability (ICC = 0.99 and ICC = 0.78, respectively) [23].

The OLST assesses static balance performance. Participants are instructed to stand barefoot on one leg, eyes open, with the arms folded their chest, for a maximum of 30 s. The time is started when participants raised their leg and is stopped when participants’ foot either contacts with the floor or touches with the other leg or participant moves the standing foot or participant’s arms leaved the trunk. Higher time reflects better static balance [24]. The OLST indicates good test-retest reliability in PD (ICC = 0.82) [25].

The TUG is commonly used to measure functional mobility. In this test, participants are required to stand up from a chair, walk 3 m, turn around, return to the chair, and sit down again. The time taken to complete the test is recorded. Lower time demonstrates better functional mobility performance [26]. The TUG shows high test-retest reliability for patients with PD [21].

The TWSTRS is originally developed to measure the severity of CD. Its scores range from 0 to 85 points, with higher score showing higher disease severity [27, 28]. The TWSTRS has acceptable interrater reliability in patients with CD (ICC = 0.69) [27].

### 2.4. Statistical analysis

All statistical analyses were performed using the Statistical Package for the Social Sciences version 17.0 (SPSS Inc., Chicago, IL, USA). Descriptive statistics were used to describe the demographic and clinical characteristics of the participants. The normality of the distribution for all variables was evaluated by the Shapiro-Wilk test. Differences in the mean values between patients with CD and healthy people were analyzed using independent t-tests. Correlations between the TIS-total and the BBS, FSST, OLST, TUG, and TWSTRS were analyzed with Pearson’s correlation coefficients (r). The degree of correlation coefficients were classified as poor (0.0–0.25), fair (0.25–0.50), moderate (0.50–0.75), or strong (0.75–1.0) [29]. The statistical significance level was set at p < 0.05.

## 3. Results

In total, 32 patients with CD (9 males, 23 females) and 32 healthy people (10 males, 22 females) were recruited for this study. There were no significant differences in age, sex, weight, and height between patients with CD and healthy people (p > 0.05 for all). Demographic and clinical characteristics of the study participants are presented in Table 1.

Compared to the healthy people, patients with CD had significantly lower scores on the TIS-total (p < 0.001) with the TIS-dynamic sitting balance subscale (p < 0.001), and TIS-trunk coordination subscale (p < 0.001) except for TIS-static sitting balance subscale (p = 0.078) (Table 2).

For the patients with CD, the values of the BBS, FSST, OLST, TUG, and TWSTRS, and their correlations with the TIS-total are presented in Table 3. The TIS-total indicated a strong correlation with BBS (r = 0.786; p < 0.001), and moderate correlation with the TUG, FSST, and OLST (r = −0.691; p < 0.001, r = −0.665; p < 0.001, and r = 0.536; p = 0.002, respectively). Additionally, no significant correlation was found between the TIS-total and TWSTRS (r = −0.294; p = 0.102).

## 4. Discussion

The main result of the study is that impaired trunk control, particularly the loss of performance in dynamic sitting balance and trunk coordination, was found for patients with CD. In addition, trunk control was associated with balance and functional mobility but no association with disease severity in CD.

Patients with CD demonstrated decreased trunk control compared to healthy people. Especially, dynamic sitting balance and trunk coordination worsened, except for static sitting balance for CD. The pathological head deviation could result from an offset of a nonsensory set point input that is required for head-on-trunk control [30]. Abnormal posturing of the head is also commonly combined with oscillatory head movements in CD [31,32]. Since the trunk stabilizes the field of vision and induces the integration of the vestibular input by functioning as a lowpass filter, it plays a key role in decreasing head oscillations [33]. This can represent that trunk stabilization is of great importance for controlling the position of the head in space. Therefore, increasing head oscillation may indicate impaired trunk control. On the other hand, patients with CD showed similarly in maintaining static sitting balance position compared to healthy people. This could indicate that postural sway did not impair in CD during static stance [8], and also static sitting could not be a challenging position enough to disturb postural stability. As a result, trunk control should be clinically taken into account in normal neurological examination and intervention protocol for patients with CD.

The strength of the neck reflexes, specifically the cervico-collic and vestibulo-collic reflexes, reflect the link between the head and trunk [34]. Deterioration of these two reflexes was reported in patients with CD [35, 36]; thus, the abnormalities in the neck and head may be accompanied by trunk impairments. Furthermore, patients with CD demonstrated deficiency in bilateral proprioceptive feedback [35], sensorimotor integration [37], and impairments in tactile, visuo-tactile temporal [38], and spatial somaesthetic discrimination [39], all of which contribute to the loss of the trunk control. It could be also noted that abnormalities in tactile and proprioceptive processing can have an impact on not only affected dystonic musculature but also on nonaffected body regions in people with CD [39–42]. Therefore, the trunk control could be negatively affected by the impaired somatosensory stimuli that can be a widespread neurophysiological characteristic in even focal dystonia such as CD.

Trunk control showed moderate to high correlations with static, dynamic, and functional balance. These results were in line with previous studies conducted on stroke [18, 43], multiple sclerosis [12, 44], and PD [13]. Not surprisingly, trunk control can be incorporated into balance because the trunk is the most inertial part of the body, particularly performing challenging postural tasks or dynamic conditions [45]. Patients with CD showed shifting from the head to the trunk in their reference segment to provide postural control [46]. Reduced postural control in CD [8] could reflect the loss of trunk control because the trunk acts as an initial reference frame for organizing postural control [45,47]. In the CD population, the ability of trunk control could help clinicians and researchers in interpreting balance performance.

There was a moderate correlation between trunk control and functional mobility. It was documented that poor mobility level was closely associated with poor trunk control in different neurological populations [48,49]. According to neurodevelopmental principles, movements of extremities proceed from proximal to distal body parts with the trunk, where the trunk has a key role in the movement control of extremities and further development of functional mobility [11,43,50]. Patients with CD had lower functional mobility level and gait velocity with increased step time, step length, and double support time than healthy people [8]. If the trunk control reduces, patients with CD have difficulty achieving normal gait because the center of gravity may not be continued normally. Probably, the reduced trunk control may make it difficult to carry out normal gait that results in poor mobility performance. This may support that increased trunk stability improves not only trunk control but also functional mobility [51–53]. Considering the fact that functional mobility is necessary to perform activities of daily living [54], it could be clinically suggested that specific treatment approaches on trunk control can improve participation in daily life by increasing functional mobility in CD.

There was no significant correlation between trunk control and disease severity. After botulinum toxin treatment, reduced disease severity with improved head posture and movements did not enhance balance performance [55,56]. In addition, several motor and sensorial parameters, which can possibly be related to trunk control, did not correlate with disease severity; for example, balance and postural control [57], impaired body concept [58], dystonic posture awareness [59], interoceptive sensitivity [60], and somatosensory temporal discrimination [61]. This indicates that the trunk control does not rely on the process, which gradually deteriorates as CD progresses.

The present study has several limitations. First, this study was a cross-sectional design; thus, the interpretation of the causal relationship between trunk control and balance, functional mobility, and disease severity was limited. Future studies should use a prospective design with pre and postintervention evaluations on the trunk control and a no-intervention comparison group to investigate the effects of trunk control intervention. All of the patients with CD were community-dwelling and could walk without physical assistance, which may restrict the generalizability of the findings. Generally, botulinum toxin is injected into the affected muscles of most of the patients with CD at 12-week intervals in the standard medical care of the disease, so the trunk control and its association with balance, functional mobility, disease severity should be assessed across the treatment cycle.

## 5. Conclusion

The results of this study demonstrated that patients with CD had impaired trunk control, especially dynamic sitting balance and trunk control, compared with healthy people. Impaired trunk control was associated with balance and functional mobility but not with disease severity. In clinical practice, the development of effective strategies for assessing and increasing trunk control should be included in the treatment programs to enhance balance and functional mobility.

## Figures and Tables

**Table 1:** Demographic and clinical characteristics of participants.

Characteristics	Patients with cervical dystonia (n = 32)	Healthy people (n = 32)	p

Age, y			
Mean ± SD	57.19 ± 14.25	58.47 ± 12.8	0.699

Sex, n (%)			

Male	9 (28.12)	10 (31.25)	0.784
Female	23 (71.88)	22 (68.75)	

Weight, kg			
Mean ± SD	73.41 ± 13.27	76.53 ± 9.84	0.289

Height, cm			
Mean ± SD	166.81 ± 9.22	168.41 ± 9.84	0.427

Disease duration, y			
Mean ± SD	11.38 ± 6.60	NA	NA

cm: Centimeter; kg: Kilogram; NA: Not applicable; SD: Standard deviation; y: Years.

**Table 2:** Comparison of trunk control between patients with cervical dystonia and healthy people.

Variable	Patients with cervical dystonia (n = 32)	Healthy people (n = 32)	p

TIS-total			
Mean ± SD	19.41 ± 2.66	22.69 ± 0.74	<0.001*

TIS-static sitting subscale			
Median (IQR)	7.00 (7.00–7.00)	7.00 (7.00–7.00)	0.078

TIS-dynamic sitting subscale			
Median (IQR)	8.00 (7.00–10.00)	10.00 (10.00–10.00)	<0.001*

TIS-trunk coordination subscale			
Median (IQR)	4.00 (3.25–5.00)	6.00 (6.00–6.00)	<0.001*

IQR: Interquartile range; TIS: Trunk impairment scale; SD: Standard deviation.

* p < 0.05.

**Table 3:** Outcome measures and their correlations with the trunk impairment scale in patients with cervical dystonia.

Variables	Patients with CD (n = 32)	Correlation with the TIS	

		r	p

BBS			
Mean ± SD	53.50 ± 1.93	r = 0.786	<0.001*

FSST			
Mean ± SD	11.14 ± 2.47	r = −0.665	<0.001*

OLST			
Mean ± SD	17.68 ± 8.06	r = 0.536	0.002*

TUG			
Mean ± SD	9.64 ± 2.42	r = −0.691	<0.001*

TWSTRS			
Mean ± SD	41.44 ± 15.76	r = −0.294	0.102

BBS: Berg Balance Scale; CD: Cervical dystonia; FSST: Four square step test; OLST: One-leg stance test; r: Pearson correlation coefficient; s: Seconds; SD: Standard deviation; TUG: Timed up and go test; TIS: Trunk impairment scale; TWSTRS: Toronto Western Spasmodic Torticollis Rating Scale.

* p < 0.05.
